# A surveillance study of patterns of reirradiation practice using external beam radiotherapy in Japan

**DOI:** 10.1093/jrr/rraa112

**Published:** 2020-12-21

**Authors:** Hideya Yamazaki, Gen Suzuki, Norihiro Aibe, Satoaki Nakamura, Ken Yoshida, Ryoongjin Oh, JReRT Group

**Affiliations:** Department of Radiology, Graduate School of Medical Science, Kyoto Prefectural University of Medicine, 465 Kajiicho Kawaramachi Hirokoji, Kamigyo-ku, Kyoto 602-8566 Japan; Department of Radiology, Graduate School of Medical Science, Kyoto Prefectural University of Medicine, 465 Kajiicho Kawaramachi Hirokoji, Kamigyo-ku, Kyoto 602-8566 Japan; Department of Radiology, Graduate School of Medical Science, Kyoto Prefectural University of Medicine, 465 Kajiicho Kawaramachi Hirokoji, Kamigyo-ku, Kyoto 602-8566 Japan; Department of Radiology, Kansai Medical University, Hirakata 573-1010, Japan; Department of Radiology, Kansai Medical University, Hirakata 573-1010, Japan; Department of Radiation Oncology, Miyakojima IGRT Clinic

**Keywords:** reirradiation, questionnaire, SBRT, IMRT, IGRT, particle therapy

## Abstract

The aim of this study was to survey the present status and patterns of reirradiation (Re-RT) practice using external beam radiotherapy in Japan. We distributed an e-mail questionnaire to the Japanese Society for Radiation Oncology partner institutions, which consisted of part 1 (number of Re-RT cases in 2008–2012 and 2013–2018) and part 2 (indications and treatment planning for Re-RT and eight case scenarios). Of the 85 institutions that replied to part 1, 75 (88%) performed Re-RTs. However, 59 of these 75 institutions (79%) reported difficulty in obtaining Re-RT case information from their databases. The responses from 37 institutions included the number of Re-RT cases, which totaled 508 in the period from 2009 to 2013 (institution median 3; 0–235), and an increase to 762 cases in the period from 2014 to 2018 (12.5; 0–295). A total of 47 physicians responded to part 2 of the survey. Important indications for Re-RT that were considered were age, performance status, life expectancy, absence of distant metastases and time interval since previous radiotherapy. In addition to clinical decision-making factors, previous total radiation dose, volume of irradiated tissue and the biologically equivalent dose were considered during Re-RT planning. From the eight site-specific scenarios presented to the respondents, >60% of radiation oncologists agreed to perform Re-RT. Re-RT cases have increased in number, and interest in Re-RT among radiation oncologists has increased recently due to advances in technology. However, several problems exist that emphasize the need for consensus building and the establishment of guidelines for practice and prospective evaluation.

## INTRODUCTION

Although the treatment modalities of surgery, chemotherapy and radiotherapy (RT) have improved survival and locoregional control for many cancer sites, in-field cancer recurrence after RT remains an obstacle [1]. Treatment options for in-field cancer recurrence after RT include surgery, systemic chemotherapy and reirradiation (Re-RT) [2, 3]. In the 20th century, with 2D radiotherapy, Re-RT was contraindicated due to the potential for lethal untoward toxicity [1]. With the installment of modern radiation technologies, which were designed using state-of-the-art imaging and informatics, Re-RT has evolved to include techniques such as intensity modulated radiation therapy (IMRT), stereotactic radiotherapy (SRT), particle therapy and brachytherapy (BT) [2, 3].

Unfortunately, there is limited high-level evidence supporting the use of Re-RT; therefore, radiation oncologists (RO) make clinical decisions regarding Re-RT based mainly on their own experiences and knowledge of the patient’s general health condition and preferences. To reduce this burden, we conducted a survey of Re-RT patterns of practice in the Kansai district of Japan in 2017 [4] and compared the results with those from a 2008 Canadian survey to examine changes across decades and between countries [5]. To continue this effort, we conducted a nationwide surveillance study in Japan to determine Re-RT patterns of practice for cases of in-field cancer recurrence after previous RT.

## MATERIALS AND METHODS

A survey questionnaire (see online supplementary material) was e-mailed to ROs registered in the Japanese Society for Radiation Oncology (JASTRO) directory as of 2019. At the time of correspondence, all the respondents were actively practicing in Japan. The questionnaire consisted of two parts.

Part 1 (for institutions) of the survey questionnaire requested information regarding the number of Re-RT cases in the years 2018, 2017 and 2016, 2008–2012 and 2013–2018 in terms of total numbers and as sub-grouped by radiation sites. We e-mailed questionnaire to JASTRO members using JASTROgram in June 2019. The number of JASTRO members was estimated at 3982 as of August 2019 [6]. The number of institutions equipped with a radiotherapy machine was 892 as of May 2016 (Japanese structure survey of radiation oncology in 2015 [7]).

In addition, we sent a questionnaire (only part 1) to several institutions equipped with special machines, which consisted of (i) tomotherapy, (ii) CyberKnife and (iii) particle beam facilities. Questionnaires were sent via the tomotherapy user meeting mailing list, CyberKnife user meeting mailing list, and institutions equipped with particle therapy. We got answers from institutions responding to the questionnaire via JASTROgram (*n* = 54), tomotherapy (*n* = 15), CyberKnife (*n* = 4) and particle beam facilities (*n* = 15).

Part 2 (for individual ROs) included indications and treatment planning for Re-RT and eight case scenarios selected from the most common tumor types (head and neck, chest, colorectal, genitourinary, gynecologic, gastrointestinal) reported in the 2008 Canadian survey and the Kansai survey [4]. The completed questionnaires were received via e-mail responded to JASTROgram.

This study defined Re-RT as RT performed after previous equivalent dose [EQD2] = 36 Gy using α/β = 3 (EQD2 = equivalent in 2 Gy fractions, i.e. RT of 30 Gy/10 fractions) or more. The biologically equivalent dose (BED) was converted into EQD2 using the linear quadratic model: EQD2 = prescription dose × (α/β + dose per fraction)/(α/β + 2), where α/β = 10 for tumors and 3 for organs at risk.

### Statistical analysis

All statistical analyses were performed using Stat-view 5.0 statistical software (SAS Institute, Inc., Cary, NC, USA). The percentage values were analyzed using the χ^2^ test, and values were compared using Mann–Whitney U analysis. All analyses used the *P* < 0.05 level of significance.

## RESULTS

### Part 1

We received replies from 85 institutions (including 13 particle beam facilities), 75 of which performed Re-RT in 2018 (88%). However, 59 institutions (78.6%) had difficulty obtaining individual Re-RT case details from their respective databases, primarily because there were no accompanying details concerning the second RT course and in-field recurrence after the initial RT. Several institutions examined the data for all patients that received RT two or more times, to identify any overlap between previous RT fields and the Re-RT field. Twenty institutions have ‘reirradiation’ as an ‘item’ or ‘tag word’ in their database system, but only nine institutions were able to extract Re-RT case information without difficulty. The responses from 37 institutions included the number of Re-RT cases (Table 1), which totaled 379 cases in 2018 (institution median = 5; 0–51, detailed distribution is shown in Supplementary Fig. 1, see online supplementary material), 568 in the period from 2009 to 2013 (3; 2–12.9) and an increase to 762 cases in the period from 2014 to 2018 (12.5; 2–225). A detailed scheme is shown in Supplementary Fig. 2, see online supplementary material. The most common Re-RT sites were the brain (*n* = 63, 17%), chest (*n* = 66, 18%), abdomen (*n* = 60, 16%), head and neck (*n* = 59, 16%) and vertebral body (*n* = 40, 11%) in 2018. From 2009 to 2013, the figures were distributed as follows: brain = 23%, chest = 24%, head and neck = 14%, abdomen = 13% and vertebral body = 17%; from 2014 to 2018, they were as follows: brain = 19%, chest = 25%, head and neck = 17%, abdomen = 11% and vertebral body = 9% (*P* = 0.0003; Table 1). Several changes in clinical pattern were found when these data were compared to those of the Kansai survey from 2017 [4]. For example, the percentage of Re-RT applied to the trunk (chest + abdomen + pelvis) increased from 37% (Kansai study) to 43%, whereas that of conventional areas (brain + bone metastases including vertebrae) decreased from 45% (Kansai study) to 38% (not in row number). Six particle-beam therapy facilities also reported the number of Re-RT cases, which indicated a similar growing trend: 110 cases were found in the period from 2009 to 2013 (0; 0–88) and 138 were found in the period from 2014 to 2018 (2; 0–102) (Supplementary Table 1, see online supplementary material).

**Table 1:** Patient numbers treated during 2014–2018 and 2009–2013 or single years 2016, 2017 and 2018

**(a) Patient numbers treated during 2014–2018 and 2009–2013**							
	2009–2013	(%)	2014–2018	(%)	*P*-value		
	(5 years: institution number = 23)		(5 years: institution number = 23)				
Total number	508		762				
Institution median (min–max)	3 (0–235)		12.5 (0–295)				
Brain	95	(23%)	129	(19%)	0.0003		
Head and neck	58	(14%)	112	(17%)			
Chest	80	(19%)	163	(25%)			
Abdomen	54	(13%)	76	(11%)			
Pelvis	22	(5%)	47	(7%)			
Bone metastasis	19	(5%)	40	(6%)			
Vertebrae metastasis	70	(17%)	57	(9%)			
Other	21	(5%)	38	(6%)			
**(b) Patient numbers treated during single years 2016–2018**							
	**2016**	**(%)**	**2017**	**(%)**		**2018**	**(%)**
	**(single year, institution** **number = 36)**		**(single year, institution number = 38)**			**(single year, institution** **number = 37)**	
Total number	361		359			379	
Institute median (min–max)	3.5 (0–75)		5 (0–72)			5 (0–51)	
Brain	64	(18%)	87	(24%)		63	(17%)
Head and Neck	61	(17%)	40	(11%)		59	(16%)
Chest	60	(17%)	76	(21%)		66	(18%)
Abdomen	46	(13%)	56	(16%)		60	(16%)
Pelvis	30	(8%)	26	(7%)		28	(8%)
Bone metastasis	32	(9%)	23	(6%)		29	(8%)
Vertebrae metastasis	30	(8%)	27	(8%)		40	(11%)
Other	15	(4%)	18	(5%)		28	(8%)

Summation of each number does not always equal to total number; several institutions report only ReRT number in a single year.

### Part 2

#### Respondent demographics and Re-RT eligibility and exclusion criteria

In total, 47 physicians responded to part 2 of the survey. The median of years of experience was 20 (3–37) years. Of the total respondents, 48% had been in practice for >20 years, whereas 2% had been in practice for <5 years. More experienced physicians replied to this questionnaire than to the previous 2017 Kansai survey (Table 2) [4]. Respondents were asked to comment on patient factors that would influence their decision to recommend Re-RT. Important factors included age (72%), performance status (72%), life expectancy (88%), absence of distant metastases (66%) and time interval since previous treatment (70%) (Table 3). Most of the respondents believed that patients should have a minimum Eastern Cooperative Oncology Group performance status of 2, a minimum life expectancy of 6–12 months, and an interval of 6 months or more from the initial treatment to be considered for Re-RT (Table 3). These figures were similar to those found in the Kansai survey [4].

**Table 2:** Demographic data of ROs

		No. of ROs	ROs (%)	No. of ROs in Kansai survey [4]	ROs in Kansai survey (%) [4]	*P*-value
Experience in RO practice, years	<5	1	(2%)	7	(21%)	0.0108
	5–10	8	(17%)	8	(24%)	
	11–20	15	(31%)	11	(32%)	
	>20	23	(48%)	8	(24%)	

**Table 3:** Factors affecting indication of re-irradiation

		No. of ROs	(%)	No. of ROs in Kansai survey [4]	(%)	*P*-value	Detailed factor	No. of ROs	(%)	No. of ROs in Kansai survey [4]	(%)	*P*-value
Age	No	12	(26%)	12	(35%)	n.s.						
	Yes	34	(72%)	22	(65%)							
	Unc	1	(2%)	0	(0%)							
Performance status	Yes	34	(72%)	25	(83%)	n.s.	ECOG 1≥	7	(19%)	1	(4%)	0.0641
	No	12	(26%)	6	(20%)		ECOG 2≥	16	(44%)	13	(50%)	
	Unc	1	(2%)	0	(0%)		ECOG 3≥	6	(17%)	6	(23%)	
							ECOG 4≥	6	(17%)	0	(0%)	
							Unc	1	(3%)	6	(23%)	
Life expectancy	Yes	49	(88%)	21	(70%)	n.s.	1–3 months	12	(32%)	2	(8%)	n.s.
	No	6	(11%)	9	(30%)		3–6 months	10	(26%)	9	(38%)	
	Unc	1	(2%)	1	(4%)		6–12 months	11	(29%)	7	(29%)	
							1–2 years	2	(5%)	4	(17%)	
							≥2 years	2	(5%)	1	(4%)	
							Unc	1	(3%)	1	(4%)	
Distant metastasis	Yes	31	(66%)	22	(67%)	n.s.						
	No	14	(30%)	11	(33%)							
	Unc	2	(4%)	0	(0%)							
Disease free interval after initial RT	Yes	33	(70%)	22	(73%)	n.s.	1–3 months	0	(0%)	0	(0%)	n.s.
	No	14	(30%)	8	(27%)		3–6 months	7	(21%)	4	(15%)	
	Unc	0	(0%)	1	(4%)		6–12 months	20	(59%)	12	(46%)	
							1–2 years	4	(12%)	3	(12%)	
							≥2 years	2	(6%)	0	(0%)	
							Unc	1	(3%)	7	(27%)	

Summation of % dose not equal 100% because of duplicated answers. n.s. = not siginificant, Unc = uncertain.

#### Factors affecting Re-RT treatment planning

A total of 58% of ROs agreed that a metastatic workup was necessary before considering Re-RT (Table 4). Most of the ROs preferred to use summation BED (62%) or EQD (67%) with an α/β = 2–3 rather than the summation of the irradiated dose (27%). Significant factors used to determine the Re-RT dose were as follows: previous dose (58%), summation of irradiated dose (29%), summation of BED (62%), summation of EQD (62%), irradiated volume (64%) and clinical decision-making factors (96%). The most important factors were the clinical decision-making factors (96%) and the summation of EQD (24%). Sixty-four percent of ROs simultaneously prescribed chemotherapy and Re-RT. Similar responses were observed in the Kansai survey [4], except there was an increased use of chemotherapy in the present study. Thirty-eight institutions were able to make a summation of dose distribution using the fusion technique of repeated radiotherapy plans (83%) to perform dose volume analysis. According to the 46 respondents who reply the number of maximal ReRT experience, the mean number of times repeated radiotherapy was 3 (1–7).

**Table 4:** Factors affecting Re-RT planning

		No. of ROs	(%)	(%) of Kansai survey [4]
Metastatic work up	Yes	26	(58%)	(69%)
	No	18	(40%)	(13%)
	Unc	1	(2%)	(19%)
Normal tissue tolerance	Summation of irradiated dose	12	(27%)	(91%)
	Summation of BED	28	(62%)	(68%)
	Summation of EQD	30	(67%)	NA^c^
Factor to decide Re-RT dose	Previous dose	26	(58%)	(74%)
	Summation of irradiated dose	13	(29%)	(79%)
	Summation of BED	28	(62%)	(79%)
	Summation of EQD	28	(62%)	NA
	Irradiated Volume	29	(64%)	(38%)
	Clinical decision	43	(96%)	(79%)
Most important factor to decide Re-RT dose^a^	Previous dose	4	(9%)	
	Summation of irradiated dose	4	(9%)	
	Summation of BED	7	(16%)	
	Summation of EQD	11	(24%)	
	Irradiated volume	2	(4%)	
	Clinical decision	19	(42%)	
	Other^b^	2	(4%)	
Chemotherapy use	Yes	29	(64%)	(16%)
	No	13	(29%)	(50%)
	Unc	6	(13%)	(34%)

^a^Summation of % dose not equal 100% because of duplicated answers; ^b^distance from organ at risk, patients will etc.; Unc = uncertain, NA = not available.

#### Responses to site-specific case scenarios

The ROs were presented with eight different clinical scenarios (see online supplementary material) and asked a series of questions regarding how they would manage them. Responses to the site-specific questions are summarized in Table 5. In general, >60% of the ROs recommended Re-RT for every scenario, which was similar to the trend observed in the Kansai survey [4].

**Table 5:** Attitude to Re-RT in case scenarios

Case No.		ROs answer				Kansai survey [4]	Methods and dose fractionation (in descending order)
		Yes	No	Unc^a^	Yes (%)	Yes (%)	ROs answer
1	Central nervous system	35	6	0	(85%)	(94%)	SRI (25–36 Gy/4–9 fr^a^, 8–20 Gy/1 fr, 30–50 Gy/10 fr), IMRT/3D-CRT (60 Gy/20 fr, 30 Gy/10 fr, 36 Gy/6–9 fr + Bev^a^, 40–50 Gy/20–25 fr), BT (36 Gy/6 fr), BNCT
2	Head and neck cancer (nasopharyngeal cancer)	34	8	0	(81%)	(87%)	IMRT (50–70 Gy/25–35 fr, 42 Gy/12 fr, 20 Gy/10 fr + HT^a^), SRI (30–42 Gy/5–8 fr, 40–50 Gy/10–20 fr), 3D-CRT (30 Gy/25 fr bid^a^), BT (24 Gy/4 fr), CP^a^
3	Non-small-cell lung cancer	31	10	0	(76%)	(72%)	3D-CRT (45 Gy/15 fr, 20–30 Gy/10 fr, 50 Gy/25fr, 30 Gy/25 fr bid), IMRT (40–60 Gy/10–30 fr, 31 Gy/8 fr, 30 Gy/10 fr, 50.4 Gy/28 fr + HT), SRI (30–40 Gy/5–10 fr), CP
4	Breast cancer	25	16	0	(61%)	(66%)	3D-CRT (40–60 Gy/20–30 fr), IMRT (30–50 Gy/10–25 fr), 2D (40–50 Gy/16–25 fr), electron (35–60 Gy/10–30 fr), SRI
5	Brain metastasis (breast)	27	14	1	(64%)	(61%)	SRI (50 Gy/16 fr, 18–22 Gy/1 fr, 20–50 Gy/8–10 fr, 30–36 Gy/3–5 fr), 3D-CRT WB^a^ (15–25 Gy/5–10 fr), IMRT
6	Cervical cancer	39	2	0	(95%)	(82%)	BT (18–50 Gy/4–10 fr), IMRT (40–50 Gy/10–20 fr) + BT (16–20 Gy/1–5 fr), IMRT (60 Gy/30 fr)
7	Rectal cancer	37	5	0	(88%)	(65%)	IMRT (50 Gy/25 fr, 20 Gy/5 fr, 30–50 Gy/5–10 fr, 50–65 Gy/20–25 fr), 3D-CRT (25–30 Gy/10 fr, 8 Gy/1 fr), CP (70 Gy/35 fr, 70.4 Gy/16 fr), BT (30–42 Gy/5–10 fr)
8	Prostate cancer	39	3	0	(93%)	(84%)	IMRT (20–40 Gy/5–20 fr), 3D-CRT (8 Gy/1 fr, 15–30 Gy/3–10 fr), SRI (8 Gy/1 fr, 30 Gy/5–10 fr), 2D (4 Gy/1 fr), BT

^a^CP = charged particle, Unc = uncertain, bid = twice in a day, HT = hyperthermia, WB = whole brain, Bev = bevacizumab, fr = fraction.

***Case 1: central nervous system cancer.*** A 55-year-old male with a second local recurrence of anaplastic astrocytoma after primary surgery, postoperative RT (60 Gy/30 fractions), and a second surgery with temodazole treatment. Eighty-five percent of ROs agreed to treat this patient with Re-RT, and stereotactic irradiation (SRI) was the main technique chosen for Re-RT in this case (SRI: 28 [80% of those who responded ‘Yes’ to Re-RT treatment]; IMRT: 13; 3D conformal radiotherapy [3D-CRT]: 3; BT: 1; boron neutron capture therapy [BNCT]: 1).

***Case 2: head and neck cancer (nasopharyngeal cancer).*** A 45-year-old male with local recurrence (3 × 2 cm) 3 years after chemoradiotherapy (70 Gy/35 fractions). Eighty-one percent of ROs agreed to treat with Re-RT, and IMRT was the main technique chosen in this case (IMRT: 21 [62%]; SRI: 15; 3D-CRT: 2; BT: 1; charged particle: 1).

***Case 3: lung cancer.*** A 63-year-old male presented with a 3-week history of hemoptysis due to local recurrence of stage IIIA non-small-cell lung cancer after chemoradiotherapy (60 Gy/30 fractions) 16 months previously. Seventy-six percent of ROs agreed to treat with Re-RT, and 3D-CRT was the primary technique chosen in this case (3D-CRT: 19 [61%]; IMRT: 13; SRI: 7; charged particle: 1).

***Case 4: breast cancer.*** A 52-year-old female with a history of T2 N1 M0 invasive ductal cancer 7 years previously who had undergone right modified radical mastectomy followed by chemotherapy and 5 weeks of chest wall and regional nodal irradiation at 50 Gy/25 fractions, now presenting with a positive microscopic deep resection margin after surgical excision for ulcerative scar recurrence measuring 4 × 3 cm. Sixty-one percent of ROs agreed to treat with Re-RT, and the preferred technique selected was 3D-CRT for this case (5 electron 3D-CRT: 14 [56%]; 5 electron IMRT: 6; 2D planning radiotherapy: 4; SRI: 1).

***Case 5: metastatic central nervous system.*** A 61-year-old female with breast cancer and multiple (>10) brain metastases after whole brain radiotherapy 6 months previously (30 Gy/10 fractions) presenting with headache and dizziness. Sixty-four percent of the ROs agreed to treat with Re-RT, and SRI was selected as the primary technique in this case (SRI: 23 [85%]; IMRT: 2; 3D-CRT: 3).

***Case 6: gynecological cancer.*** A 59-year-old female with cervical cancer who received surgery and postoperative radiotherapy (45 Gy/25 fractions), presenting with low volume vaginal vault relapse. In this case, nearly all ROs (95%) reported they would offer Re-RT, and BT was the primary choice of treatment (BT: 37 [95%]; IMRT: 9; 3D-CRT: 3).

***Case 7: rectal cancer.*** A 66-year-old male with presacral recurrence 2 years and 3 months after neoadjuvant chemoradiotherapy with 50.4 Gy/28 fractions and surgery for rectal cancer, presenting with progressive vague pain in the sacral area and a recent history of left foot drop. Eighty-eight percent of ROs agreed to treat with Re-RT, and IMRT was the primary technique chosen for this case (IMRT: 27 [73%]; 3D-CRT: 9; charged particle: 7; BT: 4).

***Case 8: prostate cancer.*** A 72-year-old male with recurrent spinal vertebrae metastasis (Thoracic spine level) 13 months after radiotherapy (30 Gy/10 fractions), presenting with lower limb weakness. Ninety-three percent of ROs agreed to Re-RT, and IMRT was the primary choice of treatment for this case (IMRT: 21 [54%]; 3D-CRT: 15; SRI: 4; 2D: 1; BT: 1).

For these site-specific scenarios, 61–95% of ROs recommended Re-RT, and most (95%) of the ROs recommended Re-RT-BT for the cervical cancer case, while relatively few (61%) ROs recommended Re-RT-BT for the breast cancer case. This 95% recommendation was higher than the 82% found in the Kansai survey [4]. Newer technologies, such as 3D-CRT, IMRT, SRI, SRT, charged particle therapy, hyperthermia and BNCT, have influenced Re-RT planning and facilitated an increased potential for normal tissue tolerance. For instance, seven ROs in this survey recommended charged-particle radiotherapy for rectal cancer.

## DISCUSSION

This study was a national survey that aimed to determine the present state of Re-RT and compare trends and attitudes toward Re-RT among Kansai ROs in 2015 with those in the present [4]. More than 80% of the responding institutions performed Re-RT this past decade, and the numbers have increased from 508 in the period from 2009 to 2013 (institution median = 3; 2–235) to 762 in the period from 2014 to 2018 (12.5; 0–295), indicating that ROs are becoming more familiar with Re-RT. The primary site of treatment also changed slightly from the brain and vertebrae in 2017 to the chest and the head and neck in this study. In the Kansai survey from 2017, respondents from 19 institutions that could actively perform Re-RT revealed a similar upward trend from the period from 2004 to 2009 to the period from 2010 to 2014 [4]. Additionally, Joseph *et al*. reported a survey of Canadian ROs in 2008 [5], showing that the estimated number of patients with in-field cancer recurrence per RO per year was 10 on average (0–15).

The growing number of Re-RTs may be due to the advancement of RT technology during the past decade, as evidenced by more patients receiving Re-RT with state-of-the-art equipment, such as SRT, IMRT, particle therapy and BT [2, 3]. Advances in external RTs with IMRT and image-guided radiation therapy (IGRT) have significantly reduced toxicities and improved outcomes. In general, Re-RT (with or without chemotherapy) has been successfully performed without excessive morbidity [2, 3].

Re-RT can be instilled in almost all in-field local recurrent tumors, such as the brain [8, 9], head and neck [10, 11], lungs [12], breast [13], abdomen [14], pelvis, prostate and gynecological regions [15–18], bone [19] or secondary primary cancers [10]. We know that the patterns of disease progression and prognosis are largely dependent on the site and cancer histopathology. We can anticipate a long life expectancy for breast [13] or prostate cancer [15], for instance, allowing for a curative approach to maximize a sustained quality of life. However, head and neck [8, 9], brain [8, 9] and pancreatic cancers [14] often have grim prognoses; therefore, we predominantly use a palliative approach to avoid toxicity, which hinders active management.

Adequate risk assessment before Re-RT of potential complications in normal tissue is difficult because there is a lack of sufficient data on recovery from radiation injury. Acute reactions may improve within a few months, and retreatment or irradiation of untreated tissue has been shown to be tolerated [20]. However, late-responding tissues have shown a different pattern of recovery from radiation organ by organ. The heart, bladder and kidney do not demonstrate long-term recovery, whereas organs such as the skin, mucosa, lungs and spinal cord do [18]. The possibility of unacceptably high severe toxicity, even lethal catastrophic arterial rupture, carotid blow-out syndrome [21] or rupture of the thoracic aorta [22], should be anticipated. Recently, a few dose volume constraints for each organ at risk from Re-RT have been proposed [23, 24], one of which included considering temporally-based dose recovery parameters, or ‘discount’ factors with time [23].

When considering Re-RT, ROs must make difficult clinical decisions, which depend mainly on their own experience due to the lack of high-level evidence for Re-RT. There are several considerations that could be drawn from this study to help in decision-making, such as (i) normal tissue tolerability [23, 24], (ii) availability of technical data from the first RT, (iii) palliative or radical intent relating to prognosis, and (iv) Re-RT schedule and dose fractionation-volume relationship. Such treatment decisions must be made only by multidisciplinary teams with expertise in radiotherapy and implemented on carefully selected patients. Paradis *et al*. proposed a consultation team of medical physicians in a Re-RT case [24].

Future studies involving quantitative risk analyses for each organ should be performed using image fusion (deformative technique required at times) of dose distribution in repeated radiotherapy.

In this analysis, more experienced physicians replied compared to the previous Kansai study (Table 2) [4]. Factors determining indications for Re-RT included performance status, age, life expectancy, the presence of distant metastases (other active lesions) and the disease-free interval since the initial RT. Factors affecting Re-RT planning were metastatic work-ups, summation of BED or EQD for tumor and normal tissue, and chemotherapy use, among others. Of these, the most important factor was the clinical decision-making factor, which included the patient’s general status and preference in addition to the socio-economic situation. The intent of Re-RT (radical or palliative) is also an important determinant, which is influenced by response to initial treatments, clinically apparent late effects from previous RT, performance status and the existing literature. Consensus on palliative or curative intent has also been changing recently, as illustrated by the oligometastasis concept [25].

Dose volume and fractionation relationships are important in Re-RT, though a consensus has not been reached due to the paucity of data. Alternation fractionation, such as a hyper-fractionated schedule or a day-after-day schedule, has been proposed as a means of limiting retreatment toxicity [26]. Newly installed technologies (SRT, IMRT, IGRT, particle therapy and image guided-BT) have also enhanced the potential of Re-RT; however, several concerns have simultaneously arisen that clarify the need for consensus building and the establishment of guidelines for Re-RT. Charged-particle therapy, for example, was recommended for several recurrence sites in this survey (Table 5), which was not observed in the 2008 Canadian survey [5].

Most institutions encountered difficulty in retrieving Re-RT case information from their databases. A simple system of retrieval of Re-RT cases is essential for the future exploration of the utility of Re-RT. Our survey revealed the need for the item or tag word ‘reirradiation’ to be available in the database systems to facilitate the easy retrieval of Re-RT cases. At present, standard databases, including the JASTRO structure survey, did not have a tag word ‘reirradiation’. A definition of ‘reirradiation’ should also be discussed for consensus building. In addition, guidelines should be established for practice and prospective evaluations, since they varied from physician to physician and depended on each patient’s background and the equipment available at each institution.

This manuscript included several limitations. Firstly, this study included a small number of Japanese institutions (maybe <10%) and therefore did not present the status of the whole of Japan. Next, although we compared outcomes between the previous Kansai survey and the present study, the treatment environment in the the Kansai region and Japan cannot be said to be the same, therefore, the difference in background should be kept in mind. In addition, there may be more unreported ReRT cases that were not detected because of an insufficient database system. Finally, institutions that responded to this survey might perform a lot of re-irradiation and may have an aggressive policy about re-irradiation, while institutions that did not respond might have a conservative policy for re-irradiation and rarely perform re-irradiation, which implies a potential bias.

In conclusion, Re-RT cases have increased in number, and interest in Re-RT among radiation oncologists has increased recently due to advances in technology. However, several problems exist that emphasize the need for consensus building and the establishment of guidelines for practice and prospective evaluation.

## Supplementary Material

reRT_supplementary_table_submit_rraa112

Supplemental_Fig_1_submit_rraa112
