# Supplementary material for: A surveillance study of patterns of reirradiation practice using external beam radiotherapy in Japan
Source: J Radiat Res. 2020 Dec 21;62(2):285–93. doi: 10.1093/jrr/rraa112 (PMC7948832; doi:10.1093/jrr/rraa112)
Supplement: Supplemental_Fig_1_submit_rraa112 [file supplemental_fig_1_submit_rraa112.pptx]

## Slide 1
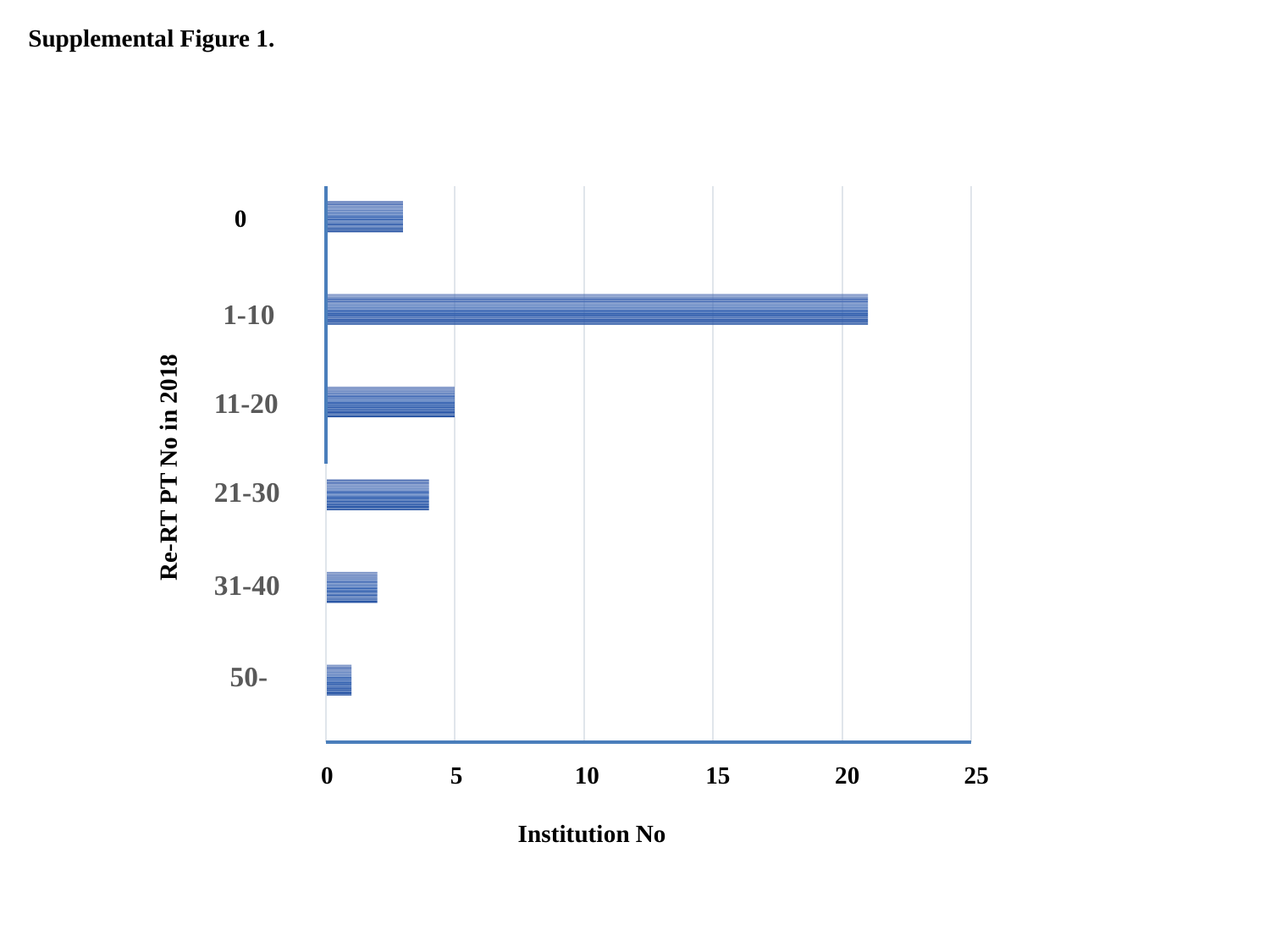

Supplemental Figure 1.
0
5
10
15
20
25
0
1-10
Re-RT PT No in 2018
11-20
21-30
31-40
50-
Institution No

## Slide 2
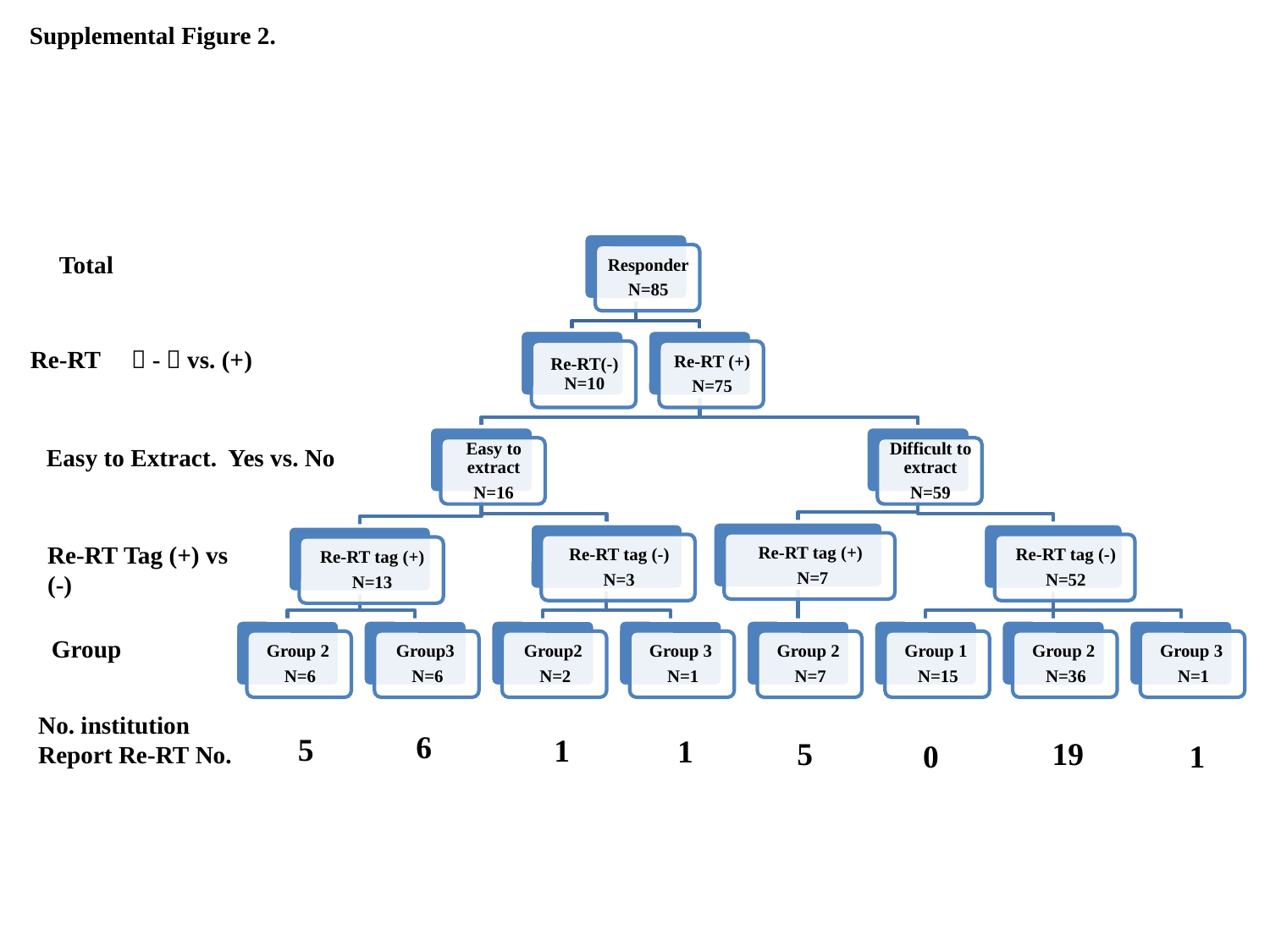

Supplemental Figure 2.
Total
Re-RT　（-）vs. (+)
Easy to Extract. Yes vs. No
Re-RT Tag (+) vs (-)
Group
No. institution
Report Re-RT No.
6
5
1
1
5
19
0
1
